# Environmental Medicine : Blood Test to Detect Lung Cancer

**Published:** 2006-12

**Authors:** Victoria McGoverns

Lung cancer kills more Americans each year than all leukemias and cancers of the breast, prostate, and ovary combined. But a new diagnostic that may allow early detection of the dominant form of the disease could change that grim picture. A team of researchers at the University of Kentucky Chandler Medical Center in Lexington led by Li Zhong, an assistant professor in the Division of Pulmonary, Critical Care, and Sleep Medicine, has developed a blood test for detecting non–small cell lung cancer (NSCLC), the disease associated with 80% of diagnosed lung cancers.

An estimated 175,000 new cases of lung cancer will be diagnosed in 2006, according to the American Cancer Society, and about 162,000 people will die of the disease. Fewer people are dying of NSCLC than in the past, which may reflect fewer people smoking. Still, those diagnosed tend to be diagnosed late, and those diagnosed late tend to die of the disease.

Spiral computed tomography (CT) imaging is today’s gold standard for lung cancer detection, able to identify tumors less than 1 cm in diameter. But at more than $400 a test, spiral CT imaging is far too costly for population-level screening. Further, there is growing concern that spiral CT imaging is overdetecting lung cancers that, left alone, would not progress, and might even regress. A better test would be one specific for cancers that are actively progressing.

Zhong’s test, described in the July 2006 issue of the *Journal of Thoracic Oncology*, uses a panel of five cancer-associated protein markers identified by their reactivity to antibodies in cancer patients’ blood. The markers were identified by generating a panel of proteins corresponding to genes expressed in NSCLC cells, then probing them with antibodies from NSCLC patients’ blood. The five proteins that were the most discriminating—reacting to antibodies from patients much more than they reacted to antibodies found in the blood of nonpatients—were tested together for their ability to discern between blood samples drawn from diagnosed NSCLC patients and those from nonpatient controls.

Used together, the five markers form a “fingerprint” for cancerous samples, with a readout that is highly specific, able to separate cancer and noncancer samples in 87.5% of cases tested. By comparison, the widely hailed prostate-specific antigen test for diagnosing prostate cancer is just 36% accurate, and the CA125 test for ovarian cancer is 57% accurate. The new test can also recognize cancerous samples in earlier stages.

The cost of the new test will depend on the diagnostic platform ultimately developed, but is expected to be significantly less than the current standard. “We can lower the risk of people [progressing] to advanced-stage lung cancer,” Zhong says. “If you can screen less expensively first, then you can suggest some people for follow-up—come back in six months and redo the test, or for some, go ahead and take the CT test now.”

Jonathan Cohen, whose Rockville, Maryland, company 20/20 GeneSystems is developing the new test toward clinical application, is confident that the test could change outcomes. “A lot of diagnostic products stumble because they won’t make a clear clinical impact,” he says. “This is one where there’s a significant unmet need and a clear and very compelling clinical utility.”

Ruth Etzioni, a cancer statistician at the Fred Hutchinson Cancer Research Center in Seattle, recommends caution, though. Of this and the other new fingerprint diagnostics coming into use, she says, “The sensitivity and the ability to identify tumors is only the first step. Then whether it actually carries with it a significant benefit and a low harm profile is the key.”

## Figures and Tables

**Figure:**
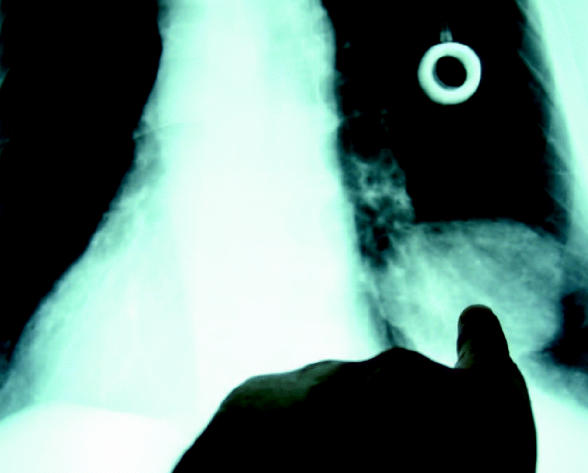
Pinpointing lung cancer A new blood test may help doctors detect non–small cell lung cancers earlier.

